# Dysregulation of the Glycolytic Entry Gate and Survival Route by Advanced Lipoxidation End Products in Diabetes Mellitus

**DOI:** 10.1155/joph/8316457

**Published:** 2026-07-29

**Authors:** Anwesa Mondal, Subhasish Pramanik, Chiranjit Bose, Asif Mondal, Lakshmi Kanta Mondal

**Affiliations:** ^1^ Department of Ophthalmology, Regional Institute of Ophthalmology, Medical College & Hospital Campus, Kolkata, West Bengal, India; ^2^ Department of Endocrinology & Metabolism, Institute of Post Graduate Medical Education & Research and SSKM Hospital, Kolkata, West Bengal, India

**Keywords:** diabetic retinopathy, hexanoyl-lysine, hexokinase, lactate dehydrogenase

## Abstract

**Background:**

Diabetic retinopathy (DR), a major microvascular complication of Type 2 diabetes mellitus (T2DM), is linked to oxidative and lipoxidative stress–induced metabolic dysfunction. Advanced lipoxidation end products (ALEs) cause metabolic abnormalities, and hexanoyl‐lysine (HLY) is a well‐known ALE. ALEs may interfere with glycolytic enzymes and disrupt cellular redox balance. Hexokinase (HK), the entry enzyme of glycolysis, and lactate dehydrogenase (LDH), crucial for metabolic survival under hypoxia, may be particularly susceptible to ALE‐induced alterations. Nonetheless, the association between circulating ALEs and these glycolytic checkpoints, as well as redox status across DR stages, remains unclear.

**Methods:**

A total of 236 consecutively selected subjects were enrolled, including healthy controls (HC, *n* = 57), T2DM without DR (DNR, *n* = 59), mild nonproliferative DR (MNPDR, *n* = 55), and high‐risk proliferative DR (HRPDR, *n* = 65). Plasma HLY was measured by using enzyme‐linked immunosorbent assay. Erythrocyte HK activity, plasma LDH activity, and the ratio of the oxidized to reduced forms of nicotinamide adenine dinucleotide (NAD^+^/NADH) were determined using standard biochemical methods.

**Results:**

HLY levels increased progressively from HC to HRPDR. The HK activity and the NAD^+^/NADH ratio decreased, while LDH increased across groups. In univariate regression analysis, HLY showed a negative association with HK and the NAD^+^/NADH ratio and a positive association with LDH. However, in multivariate regression analysis adjusted for confounders, HLY remained independently associated only with HK, while its associations with LDH and the NAD^+^/NADH ratio were not statistically significant.

**Conclusion:**

An increase in ALE burden is associated with dysregulation of the glycolytic entry enzyme (HK) and concurrent alterations in LDH activity, which may reflect metabolic adaptation under stress conditions, along with changes in redox status across the stages of DR. The independent association between HLY and HK suggests a link between lipoxidative stress and impaired glycolytic initiation, which may represent a metabolic alteration associated with the progression of DR.

## 1. Introduction

Diabetic retinopathy (DR) is among the most prevalent microvascular complications associated with Type 2 diabetes mellitus (T2DM) and remains a major cause of visual impairment worldwide [1]. Chronic hyperglycemia has long been recognized as a key contributor to DR onset. However, recent evidence suggests that lipid peroxidation associated with dyslipidemia also plays a significant role in aggravating microvascular injury in T2DM [2]. Advanced lipoxidation end products (ALEs), toxic lipid‐derived compounds, are believed to disrupt glycolytic regulation by modifying critical glycolytic enzymes, leading to biochemical alterations that promote DR progression [3]. In this context, glycolysis may be viewed as a regulated metabolic cascade governed by key checkpoints, where specific enzymes regulate metabolic continuity during cellular stress. Maintenance of glycolysis—the principal pathway of glucose utilization—may, therefore, help delay or prevent diabetic complications [3].

The glycolytic pathway begins with phosphorylation of glucose to glucose‐6‐phosphate (G6P), a central metabolite linking multiple metabolic pathways. During glycolysis, G6P undergoes further metabolism through rate‐limiting steps dependent on oxidized nicotinamide adenine dinucleotide (NAD^+^). Persistent hyperglycemia reduces NAD^+^ availability, resulting in metabolic disturbances and impaired intracellular glucose utilization [4, 5]. Such disturbances may adversely affect both upstream glucose entry mechanisms and downstream compensatory metabolic routes.

Hexokinase (HK), the first and rate‐limiting enzyme of glycolysis, regulates intracellular glucose utilization by catalyzing the initial committed step of glucose metabolism. Recent studies indicate that glucose‐mediated stabilization of HK2 protects against proteolysis and cellular dysfunction during hyperglycemia [6]. Acting as the metabolic “entry gate,” HK regulates intracellular glucose trapping and utilization. Reduced HK activity may represent an early biochemical alteration leading to glycolytic dysregulation, intracellular glucose accumulation, and metabolic stress in diabetic complications.

Under pseudohypoxic conditions, lactate dehydrogenase (LDH) sustains glycolysis by converting pyruvate and regenerating NAD^+^ from reduced nicotinamide adenine dinucleotide (NADH) [5]. LDH, therefore, functions as a metabolic “survival route” enzyme that preserves glycolytic flux during compromised oxidative metabolism. Increased lactate production associated with anaerobic glycolysis may reduce neuronal pH and impair glutamate uptake [7]. Elevated extracellular glutamate activates N‐methyl‐D‐aspartate (NMDA) receptors, increasing intracellular Ca^2+^ levels and stimulating phospholipase activity, leading to membrane phospholipid degradation and production of oxygen‐derived free radicals [8]. These reactive oxygen species (ROS) initiate lipid peroxidation, generating ALE adducts through interactions with membrane proteins [9, 10].

ALEs can alter protein structure, including glycolytic enzymes, by obstructing substrate binding at active sites [11]. Such modifications may compromise both the glycolytic entry gate (HK) and survival route (LDH), thereby disrupting glycolytic continuity and leading to intracellular glucose imbalance and metabolic dysfunction.

Our previous studies demonstrated associations between elevated advanced glycation end products (AGEs) and increased ALE levels with reduced antioxidant enzyme activities in erythrocytes across different stages of DR [12, 13]. Enzyme activity measurement in serum or plasma is widely used to assess tissue injury [14]. Serum or plasma LDH activity has been evaluated as an indicator of tissue damage and physiological stress in conditions such as myocardial infarction and acute pulmonary injury [15, 16]. Altered LDH activity in diabetes may, therefore, reflect adaptive activation of the survival route in response to impaired entry gate function and metabolic stress.

In addition to enzymatic alterations, oxidative and lipoxidative stresses disrupt intracellular redox balance, particularly the NAD^+^/NADH ratio. A declining NAD^+^/NADH ratio may reduce glycolytic efficiency, impair cellular energy generation, and increase susceptibility to metabolic injury in DR.

Despite growing evidence linking the formation of ALEs to oxidative stress and cellular dysfunction, the association between circulating ALE levels and key glycolytic enzymes across different stages of DR remains unclear. In particular, the interaction between ALE burden and the activities of HK, LDH, and the NAD^+^/NADH redox balance may provide valuable mechanistic insight into disease progression. Therefore, this cross‐sectional study aimed to investigate the association between ALE, represented by hexanoyl‐lysine (HLY), and the activities of essential glycolytic enzymes, namely HK and LDH, along with the NAD^+^/NADH redox ratio across various stages of DR.

## 2. Methodology

### 2.1. Study Subjects

This study enrolled 236 consecutively selected participants from the “Retina Clinic” of “Regional Institute of Ophthalmology” (RIO), Kolkata, consisting of 57 healthy controls (HCs), 59 T2DM subjects with no DR (DNR), 55 with mild nonproliferative DR (MNPDR), and 65 with high‐risk proliferative DR (HRPDR). Subjects aged 45–65 years were included in the present study. Individuals with coronary artery disease, hypertension (systolic blood pressure [BP] > 140 mm Hg, diastolic BP > 90 mm Hg, or undergoing antihypertensive treatment), diabetic peripheral neuropathy (DPN), diabetic nephropathy (DN), or any other ocular diseases (such as glaucoma, cataracts, branch retinal vein occlusion, optic neuropathy, or Eales disease) were excluded from the present study. DPN was assessed using the Michigan Neuropathy Screening Instrument (MNSI), and participants with an MNSI score ≥ 4 were considered to have DPN and were excluded from the study [17]. Vitamin B12 deficiency was defined based on serum vitamin B12 levels, which were quantified using a sandwich enzyme‐linked immunosorbent assay (ELISA) with commercially available kits (MyBioSource Inc., San Diego, CA, USA; Cat. No. MBS701134) following the manufacturer’s protocol, and participants with serum vitamin B12 levels < 200 pg/mL were considered vitamin B12 deficient and excluded [18]. DN was identified in participants with serum creatinine levels > 1.5 mg/dL and/or urinary albumin–creatinine ratio (UACR) ≥ 300 μg/mg, and such participants were also excluded [19]. None of the study participants were receiving insulin or lipid‐lowering medications during the study period. Individuals receiving lipid‐lowering therapy were excluded because these agents can modify lipid metabolism and potentially affect lipid peroxidation and the formation of ALEs [20, 21].

### 2.2. Ophthalmological Examinations

All study subjects underwent comprehensive ophthalmological evaluations, which included slit‐lamp biomicroscopy (using a 90‐diopter lens and a Goldman 3‐mirror lens), digital fundus photography with fluorescein angiography (to confirm DR in T2DM subjects), and spectral‐domain optical coherence tomography (SD‐OCT) to detect macular edema. Visual function was also assessed by measuring visual acuity (VA). DR was diagnosed following the modified Early Treatment Diabetic Retinopathy Study (ETDRS) guidelines [22].

### 2.3. Collection and Processing of Blood Samples

Following overnight fasting, 5 mL of venous blood was collected from each subject in ethylenediaminetetraacetic acid (EDTA) vacutainers and centrifuged at 1500 × g for 10 min at 4°C temperature to separate cellular components and plasma. Packed erythrocytes were then washed with cold phosphate buffer saline by maintaining temperature at 4°C for the estimation of erythrocytes enzyme activity and NAD^+^/NADH ratio. The levels of HbA1c (%) and the enzymatic activities of HK were assessed from hemolysate samples. Plasma samples were used to evaluate glucose levels, LDH activity, and ALE like HLY.

### 2.4. Evaluation of Glycemic Status

Fasting (FPG) and postprandial (PPG) plasma glucose levels were assessed using the colorimetric endpoint method (Trinder, 1969) with a commercially available kit (Labcare Diagnostics (India) Pvt. Ltd., Mumbai, India). Glycated hemoglobin (HbA1C) was measured by direct enzymatic assay using a Diazyme Laboratories kit (Poway, CA), as previously discussed in our earlier publication (Mondal et al.) [2].

### 2.5. Measurement of HLY

Human plasma HLY was measured using a competitive enzyme immunoassay technique with commercial kits (MyBioSource, Catalog no: MBS753480) and involved a polyclonal anti‐HLY antibody and an HLY‐HRP conjugate. The assay sample and buffer were incubated with the HLY–HRP conjugate on a precoated plate for 1 hour. After incubation, the wells were decanted, washed, and a substrate for the HRP enzyme was added, followed by another incubation at 37°C. The resulting enzyme–substrate reaction produced a blue‐colored complex, which turned yellow after the addition of a stop solution. The color intensity was measured at 450 nm using a spectrophotometer. A standard curve was generated by plotting the color intensity (O.D.) against standard HLY concentrations, allowing the interpolation of HLY concentrations in the samples.

### 2.6. Assessment of Erythrocyte HK Activity

Packed erythrocytes were lysed using cold hypotonic phosphate‐buffered saline (PBS) (pH 7.4). Hemolysate was separated by centrifugation at 500 × g for 10 min at 4°C, and the supernatant was used to assess HK activity, as reported by Ramalingam et al. [23]. A reaction mixture (5.3 mL total volume) was prepared using 1 mL of glucose (0.005 M), 0.5 mL of adenosine triphosphate (ATP) (0.072 M), 0.1 mL of magnesium chloride (0.05 M), 0.4 mL of potassium dihydrogen phosphate (0.0125 M), 0.4 mL of potassium chloride (0.1 M), 0.4 mL of sodium fluoride (0.5 M), and 2.5 mL of Tris‐HCl buffer (0.01 M, pH 8.0). After 5 min of preincubation at 37°C, 1 mL of supernatant was added to initiate the reaction. A zero‐time sample was collected, and after a 30‐min incubation at 37°C, the reaction was stopped, and the residual glucose was quantified using the O‐toluidine method. The activity of HK was expressed as μmol glucose phosphorylated/min/g Hb.

#### 2.6.1. Assessment of Plasma LDH Activity

Plasma LDH activity was determined using the DGKC method as described by Javaraiah et al., utilizing an LDH‐P kit (Cat. no. D94651, DIALAB, Neudorf, Austria) [24]. The kit contained two reagents: A (pyruvate) and B (NADH). A working reagent was prepared by mixing 10 parts Reagent A with 1 part Reagent B. Then, 1000 μL of the working reagent was incubated at 30°C for 5 min before adding 20 μL of plasma. After 1 min of incubation, absorbance was measured at 340 nm. The change in absorbance (ΔA/min) was calculated and adjusted with a temperature correction factor (10,080 for 30°C) to estimate LDH activity, expressed in U/L.

### 2.7. Measurement of NAD^+^/NADH Ratio

At first, buffy coat was carefully removed from centrifuged whole blood by gentle aspiration, and packed erythrocytes were washed with cold PBS (pH 7.4) at 4°C. Approximately 10^6^ red blood cells (RBCs) were pelleted in microcentrifuge tubes and extracted using NAD^+^/NADH Extraction Buffer supplied with the kit (BioVision Research Products, Mountain View, CA, USA). Cell extraction was performed by two freeze–thaw cycles (20 min on dry ice followed by 10 min at room temperature), followed by vortexing for 10 s. The lysates were centrifuged at 20,000 × g for 5 min, and the resulting supernatant was collected and filtered through a 10‐kDa molecular weight cutoff filter to remove enzymes present in the lysate.

Total NAD^+^ (NADt) and NADH levels were determined using an enzyme cycling reaction provided in the kit. For NADH determination, aliquots of the extracts were heated at 60°C for 30 min and then cooled to room temperature prior to assay. The reaction mixture was prepared according to the kit protocol, and the absorbance of the developed color was measured at 450 nm using a microplate reader (Bio‐Rad, Model 680). Furthermore, the NAD^+^ to NADH ratio was calculated using the following formula: NAD^+^/NADH = [NAD (*t*) − NADH]/NADH.

### 2.8. Statistical Analyses

All statistical analyses were performed using IBM SPSS Statistics (IBM Corp., Armonk, NY, USA). Continuous variables were expressed as mean ± standard deviation (SD), while categorical variables were presented as frequency (*n*).

The Kolmogorov–Smirnov test was used to assess normality of data distribution. Normally distributed variables were compared among study groups using one‐way analysis of variance (ANOVA), followed by Tukey’s post hoc test. Nonnormally distributed clinical variables, particularly glycemic parameters, were analyzed using the Kruskal–Wallis test with Dunn’s post hoc correction. Categorical variables were compared using the chi‐square test.

Biochemical parameters (HLY, HK, LDH, and NAD^+^/NADH ratio) were analyzed using one‐way ANOVA with false‐discovery rate (FDR) adjustment for multiple comparisons. Effect sizes were reported as eta‐squared (*η* ^2^) for ANOVA and Cohen’s d for pairwise comparisons.

To evaluate the association between HLY and metabolic parameters (HK, LDH, and NAD^+^/NADH ratio), both univariate linear regression and multivariate general linear model (GLM) analyses using a multivariate analysis of covariance (MANCOVA) framework were performed.

In univariate analyses, each biochemical parameter was separately regressed against HLY, and unstandardized regression coefficients (B), standardized coefficients (*β*), *p* values, and adjusted *R* ^2^ values were reported.

For adjusted analyses, a multivariate GLM‐MANCOVA model was applied because HK, LDH, and NAD^+^/NADH ratio represent related metabolic and redox outcomes associated with glycolytic dysregulation in DR. In this model, HK, LDH, and NAD^+^/NADH ratio were simultaneously included as dependent variables, while HLY was entered as the primary predictor variable.

Sex was treated as a fixed factor, whereas age, BMI, HbA1c, and duration of disease were included as covariates. Disease group was incorporated as a categorical variable using dummy coding (DNR, MNPDR, and HRPDR), with HC as the reference category.

Multivariate significance was assessed using Pillai’s Trace, which was selected because of its relative robustness to violations of covariance matrix assumptions and unequal group sizes. Following the multivariate test, parameter estimates for individual dependent variables were interpreted separately. Adjusted B with corresponding 95% confidence intervals (CI), *p* values, and adjusted *R* ^2^ values were reported for each dependent variable. Partial *η* ^2^ was reported as the measure of multivariate effect size. Multicollinearity among predictors was assessed using variance inflation factor (VIF), and no significant multicollinearity was observed (VIF < 5). A two‐tailed *p* value < 0.05 was considered statistically significant.

## 3. Results

**FIGURE 1:**
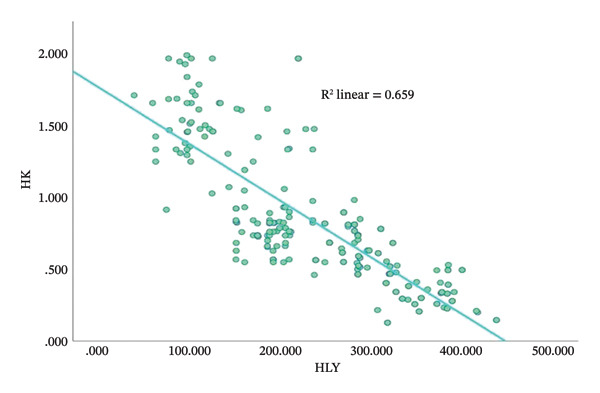
Univariate regression analysis showing the association between HK with HLY.

**FIGURE 2:**
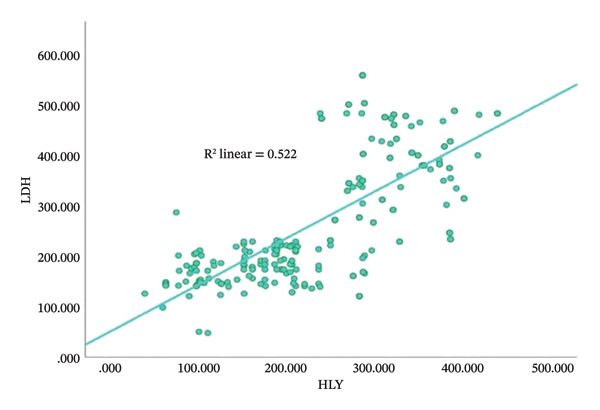
Univariate regression analysis showing the association between LDH with HLY.

**FIGURE 3:**
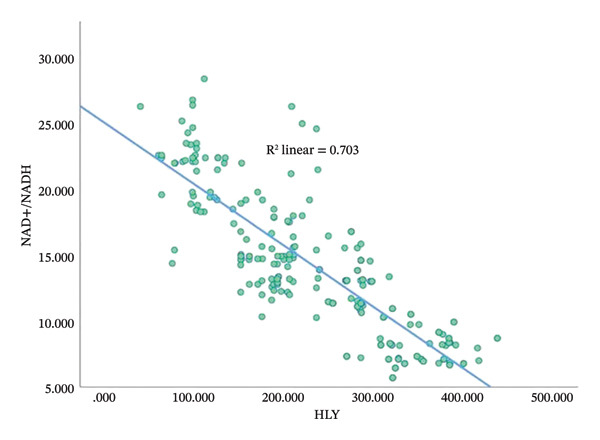
Univariate regression analysis showing the association between NAD^+^/NADH with HLY.

• The demographic and clinical parameters of the study subjects are presented in Table 1. The study showed no statistically significant differences in age, sex distribution, body mass index (BMI), or systolic and diastolic BP among the groups. However, glycemic parameters, including FPG, PPG, and HbA1c, differed significantly across the groups (*p* < 0.0001). Post hoc analysis further revealed significantly higher values in the DNR, MNPDR, and HRPDR groups compared to HC (all *p* < 0.0001), while no significant differences were observed between DNR vs. MNPDR or MNPDR vs. HRPDR.
• The concentrations of various biochemical markers showed significant differences among the four study groups (HC, DNR, MNPDR, and HRPDR) (Table 2). The HLY levels increased progressively from HC (122.98 ± 48.77) to DNR (188.24 ± 24.11), MNPDR (273.02 ± 37.32), and HRPDR (353.77 ± 37.36) groups (*p* = 1.44 × 10^−93^, *η* ^2^ = 0.847). Conversely, HK levels demonstrated a marked decreasing trend across the groups, with the highest levels observed in HC (1.54 ± 0.24) and the lowest in HRPDR (0.37 ± 0.14) (*p* = 5.30 × 10^−103^, *η* ^2^ = 0.871). LDH levels were significantly elevated across disease progression, ranging from 155.18 ± 32.23 in HC to 385.17 ± 77.03 in HRPDR (*p* = 3.25 × 10^−47^, *η* ^2^ = 0.612). Similarly, the NAD^+^/NADH ratio showed a progressive decline throughout the disease spectrum, with the highest ratio recorded in HC (21.50 ± 2.80) and the lowest in HRPDR (7.84 ± 1.10) (*p* = 5.61 × 10^−101^, *η* ^2^ = 0.866). The large effect sizes (*η* ^2^ ranging from 0.612 to 0.871) indicate substantial group‐wise differences in the studied biochemical parameters.
• Post hoc analysis of multiple comparisons indicated that HLY levels showed significant differences across all study groups (Table 3). Compared with HC, HLY levels were significantly elevated in DNR (mean difference = −65.26, FDR‐adjusted *p* = 3.74 × 10^−12^, and Cohen’s *d* = 1.82), MNPDR (−150.04, FDR‐adjusted *p* = 5.22 × 10^−25^, and *d* = 3.62), and HRPDR (−230.79, FDR‐adjusted *p* = 4.29 × 10^−35^, and *d* = 5.28). Similarly, significant increases were observed between DNR and MNPDR (−84.78, FDR‐adjusted *p* = 9.53 × 10^−16^, and *d* = 2.75) and DNR and HRPDR (−165.53, FDR‐adjusted *p* = 1.00 × 10^−27^, and *d* = 4.98), as well as between MNPDR and HRPDR (−80.75, FDR‐adjusted *p* = 2.89 × 10^−14^, and *d* = 2.31). All pairwise comparisons demonstrated large to very large effect sizes, indicating a marked and progressive increase in HLY levels with increasing severity of DR.
• Post hoc multiple comparison analysis also demonstrated that HK levels differed significantly across all study groups (Table 4). Compared with HC, HK levels were markedly reduced in DNR (mean difference = 0.77, FDR‐adjusted *p* = 3.12 × 10^−34^, and Cohen’s *d* = 3.84), MNPDR (0.86, FDR‐adjusted *p* = 1.35 × 10^−36^, and *d* = 4.29), and HRPDR (1.17, FDR‐adjusted *p* = 1.39 × 10^−44^, and *d* = 5.78). Significant decreases were also observed between DNR and MNPDR (0.09, FDR‐adjusted *p* = 4.65 × 10^−3^, and *d* = 0.64) and DNR and HRPDR (0.40, FDR‐adjusted *p* = 1.41 × 10^−15^, and *d* = 2.79), as well as between MNPDR and HRPDR (0.31, FDR‐adjusted *p* = 2.53 × 10^−13^, and *d* = 2.21). The pairwise comparisons demonstrated moderate to very large effect sizes, indicating a progressive decline in HK levels with increasing severity of DR.
• Concerning LDH levels, post hoc multiple comparison analysis revealed significant differences across the study groups (Table 5). Compared with HC, LDH levels were significantly elevated in DNR (mean difference = −42.16, FDR‐adjusted *p* = 5.89 × 10^−7^, and Cohen’s *d* = 1.45), MNPDR (−181.86, FDR‐adjusted *p* = 4.16 × 10^−20^, and *d* = 2.26), and HRPDR (−229.99, FDR‐adjusted *p* = 2.15 × 10^−23^, and *d* = 3.61). Significant increases were also observed between DNR and MNPDR (−139.70, FDR‐adjusted *p* = 1.01 × 10^−14^, and *d* = 1.84) and DNR and HRPDR (−187.83, FDR‐adjusted *p* = 7.10 × 10^−18^, and *d* = 2.92), as well as between MNPDR and HRPDR (−48.13, FDR‐adjusted *p* = 3.32 × 10^−3^, and *d* = 0.52). Overall, the pairwise comparisons demonstrated moderate to very large effect sizes, indicating progressively increased LDH levels with increasing severity of DR.
• Moreover, post hoc multiple comparison analysis revealed significantly reduced NAD^+^/NADH ratios across the study groups (Table 6). Compared with HC, NAD^+^/NADH ratios were markedly lower in DNR (mean difference = 7.04, FDR‐adjusted *p* = 2.88 × 10^−26^, and Cohen’s *d* = 2.84), MNPDR (8.78, FDR‐adjusted *p* = 8.66 × 10^−32^, and *d* = 3.69), and HRPDR (13.66, FDR‐adjusted *p* = 6.63 × 10^−45^, and *d* = 5.92). Significant decreases were also observed between DNR and MNPDR (1.74, FDR‐adjusted *p* = 4.69 × 10^−5^, and *d* = 0.98) and DNR and HRPDR (6.62, FDR‐adjusted *p* = 1.28 × 10^−26^, and *d* = 3.47), as well as between MNPDR and HRPDR (4.88, FDR‐adjusted *p* = 1.01 × 10^−18^, and *d* = 2.86). Overall, the pairwise comparisons demonstrated moderate to very large effect sizes, indicating a progressive decline in NAD^+^/NADH ratios with increasing severity of DR.
• In univariate linear regression analysis, HLY was found to be significantly associated with all three metabolic parameters like HK, LDH, and NAD+/NADH (Figures 1–3). HLY demonstrated a notable negative correlation with HK (B = −0.004, β = −0.812, and *p* < 0.001; adjusted *R* ^2^ = 0.657) and NAD+/NADH ratio (B = −0.047, *β* = −0.839, and *p* < 0.001; adjusted *R* ^2^ = 0.702), while a significant positive correlation was found with LDH levels (B = 0.931, *β* = 0.723, and *p* < 0.001; adjusted *R* ^2^ = 0.520).
• Additionally, multivariate regression analysis demonstrated a significant overall multivariate effect of HLY on the dependent variables (Pillai’s Trace = 0.050, *F* = 3.816, *p* = 0.011, partial *η* ^2^ = 0.050). HLY was independently associated with reduced HK levels (*B* = −0.001, 95% CI: −0.001 to 0.000, *p* = 0.003), whereas its associations with LDH and the NAD^+^/NADH ratio were not statistically significant. Among the covariates, age showed a significant inverse association with LDH (*B* = −1.534, 95% CI: −2.866 to −0.202, *p* = 0.024), while BMI, duration of disease, HbA1c, and sex were not significantly associated with any outcome. Compared with the reference group, DNR, MNPDR, and HRPDR were significantly associated with lower HK levels and NAD^+^/NADH ratios (all *p* < 0.001). Moreover, MNPDR and HRPDR were associated with significantly higher LDH levels (both *p* ≤ 0.001), whereas DNR showed no significant association with LDH. The regression models demonstrated good explanatory power, with adjusted *R* ^2^ values of 0.872 for HK, 0.618 for LDH, and 0.860 for the NAD^+^/NADH ratio (as shown in Table 7).

**TABLE 1:** Comparison of demographic and clinical parameters of study subjects.

Parameters	HC (*n* = 57)	DNR (*n* = 59)	MNPDR (*n* = 55)	HRPDR (*n* = 65)	*p* value
Age (years)	51.00 ± 7.48	51.02 ± 6.12	53.11 ± 1.99	52.06 ± 6.61	0.192
Sex					0.976
Male	30	30	27	30	
Female	27	29	28	35	
BMI (kg/m^2^)	23.36 ± 3.41	25.17 ± 2.31	25.07 ± 3.97	25.02 ± 6.62	0.104
Duration of DM (years)	—	10.46 ± 5.20	11.14 ± 3.92	11.36 ± 3.68	0.784
Blood pressure (mmHg)					
Systolic	123.2 ± 9.57	127.8 ± 6.01	127.6 ± 2.91	126.3 ± 4.21	0.116
Diastolic	80.71 ± 3.28	82.39 ± 4.59	80.27 ± 4.01	81.32 ± 5.11	0.435
Glycemic status					
FPG (mg/dL)	86.75 ± 7.879	147.5 ± 14.23^∗∗∗^	158.9 ± 40.32^§§§^	153.1 ± 18.59^¥¥¥^	< 0.0001
PPG (mg/dL)	115.5 ± 11.48	190.7 ± 36.88^∗∗∗^	207.5 ± 55.11^§§§^	214.60 ± 40.27^¥¥¥^	< 0.0001
HbA1C (%)	4.77 ± 0.369	7.60 ± 0.710^∗∗∗^	8.05 ± 1.19^§§§^	7.48 ± 0.82^¥¥¥^	< 0.0001

*Note:* Values are presented as mean ± SD or frequency (*n*). Normally distributed variables were analyzed using one‐way ANOVA, whereas non‐normally distributed variables, including glycemic parameters (FPG, PPG, and HbA1c), were analyzed using the Kruskal–Wallis test followed by Dunn’s post hoc test. Categorical variables were analyzed using the chi‐square test. A value of *p* < 0.05 was considered statistically significant.

^∗∗∗^HC vs. DNR, *p* < 0.0001.

^§§§^HC vs. MNPDR, *p* < 0.0001.

^¥¥¥^HC vs. HRPDR, *p* < 0.0001.

**TABLE 2:** Comparison of different biochemical parameters among different study groups.

Variables	HC (mean ± SD)	DNR (mean ± SD)	MNPDR (mean ± SD)	HRPDR (mean ± SD)	*p* value	Effect size (*η* ^2^)
HLY (nmol/L)	122.98 ± 48.77	188.24 ± 24.11	273.02 ± 37.32	353.77 ± 37.36	1.44 × 10^−93^	0.847
HK (μmol glucose phosphorylated/min/g Hb)	1.54 ± 0.24	0.77 ± 0.14	0.68 ± 0.14	0.37 ± 0.14	5.30 × 10^−103^	0.871
LDH (U/L)	155.18 ± 32.23	197.34 ± 25.23	337.04 ± 129.11	385.17 ± 77.03	3.25 × 10^−47^	0.612
NAD^+^/NADH ratio	21.50 ± 2.80	14.46 ± 1.97	12.72 ± 1.68	7.84 ± 1.10	5.61 × 10^−101^	0.866

*Note:* Data are presented as mean ± standard deviation (SD). Group comparisons were performed using one‐way analysis of variance (ANOVA). Effect size was expressed as eta squared (*η* ^2^). DNR: T2DM subjects with no diabetic retinopathy; HLY: hexanoyl‐lysine; HK: hexokinase; LDH: lactate dehydrogenase; Hb: hemoglobin; NAD^+^: oxidized nicotinamide adenine dinucleotide; NADH: reduced nicotinamide adenine dinucleotide.

Abbreviations: HC, healthy controls; HRPDR, high‐risk proliferative diabetic retinopathy; MNPDR, mild nonproliferative diabetic retinopathy.

**TABLE 3:** Pairwise multiple comparison analysis of HLY levels among study groups.

Comparison	Mean difference	Exact *p* value	FDR‐adjusted *p*	Effect size (Cohen’s d)
HC vs. DNR	−65.26	3.12 × 10^−12^	3.74 × 10^−12^	1.82
HC vs. MNPDR	−150.04	2.61 × 10^−25^	5.22 × 10^−25^	3.62
HC vs. HRPDR	−230.79	1.43 × 10^−35^	4.29 × 10^−35^	5.28
DNR vs. MNPDR	−84.78	7.94 × 10^−16^	9.53 × 10^−16^	2.75
DNR vs. HRPDR	−165.53	5.02 × 10^−28^	1.00 × 10^−27^	4.98
MNPDR vs. HRPDR	−80.75	2.41 × 10^−14^	2.89 × 10^−14^	2.31

*Note:* Mean differences represent pairwise comparisons of HLY levels between groups obtained from Tukey’s post hoc test following one‐way ANOVA. Reported *p* values were further adjusted using the false‐discovery rate (FDR) correction method for multiple comparisons. Effect size was expressed as Cohen’s d.

**TABLE 4:** Pairwise multiple comparison analysis of HK levels among study groups.

Comparison	Mean difference	Exact *p* value	FDR‐adjusted *p*	Effect size (Cohen’s d)
HC vs. DNR	0.77	2.08 × 10^−34^	3.12 × 10^−34^	3.84
HC vs. MNPDR	0.86	6.77 × 10^−37^	1.35 × 10^−36^	4.29
HC vs. HRPDR	1.17	4.62 × 10^−45^	1.39 × 10^−44^	5.78
DNR vs. MNPDR	0.09	3.88 × 10^−3^	4.65 × 10^−3^	0.64
DNR vs. HRPDR	0.4	9.42 × 10^−16^	1.41 × 10^−15^	2.79
MNPDR vs. HRPDR	0.31	2.11 × 10^−13^	2.53 × 10^−13^	2.21

*Note:* Mean differences represent pairwise comparisons of HK levels between groups obtained from Tukey’s post hoc test following one‐way ANOVA. Reported *p* values were further adjusted using the false‐discovery rate (FDR) correction method for multiple comparisons. Effect size was expressed as Cohen’s d.

**TABLE 5:** Pairwise multiple comparison analysis of LDH levels among study groups.

Comparison	Mean difference	Exact *p* value	FDR‐adjusted *p*‐value	Effect size (Cohen’s d)
HC vs. DNR	−42.16	4.91 × 10^−7^	5.89 × 10^−7^	1.45
HC vs. MNPDR	−181.86	2.08 × 10^−20^	4.16 × 10^−20^	2.26
HC vs. HRPDR	−229.99	7.15 × 10^−24^	2.15 × 10^−23^	3.61
DNR vs. MNPDR	−139.70	8.41 × 10^−15^	1.01 × 10^−14^	1.84
DNR vs. HRPDR	−187.83	3.55 × 10^−18^	7.10 × 10^−18^	2.92
MNPDR vs. HRPDR	−48.13	2.77 × 10^−3^	3.32 × 10^−3^	0.52

*Note:* Mean differences represent pairwise comparisons of LDH levels between groups obtained from Tukey’s post hoc test following one‐way ANOVA. Reported *p* values were further adjusted using the false‐discovery rate (FDR) correction method for multiple comparisons. Effect size was expressed as Cohen’s d.

**TABLE 6:** Pairwise multiple comparison analysis of NAD^+^/NADH ratio among study groups.

Comparison	Mean difference	Exact *p* value	FDR‐adjusted *p*	Effect size (Cohen’s d)
HC vs. DNR	7.04	1.92 × 10^−26^	2.88 × 10^−26^	2.84
HC vs. MNPDR	8.78	4.33 × 10^−32^	8.66 × 10^−32^	3.69
HC vs. HRPDR	13.66	2.21 × 10^−45^	6.63 × 10^−45^	5.92
DNR vs. MNPDR	1.74	3.91 × 10^−5^	4.69 × 10^−5^	0.98
DNR vs. HRPDR	6.62	8.55 × 10^−27^	1.28 × 10^−26^	3.47
MNPDR vs. HRPDR	4.88	6.72 × 10^−19^	1.01 × 10^−18^	2.86

*Note:* Mean differences represent pairwise comparisons of NAD^+^/NADH levels between groups obtained from Tukey’s post hoc test following one‐way ANOVA. Reported *p* values were further adjusted using the false‐discovery rate (FDR) correction method for multiple comparisons. Effect size was expressed as Cohen’s d.

**TABLE 7:** Adjusted multivariate GLM‐MANCOVA analysis showing the association of HLY with HK, LDH, and NAD^+^/NADH ratio.

Predictor	HK B (95% CI)	*p* value	LDH B (95% CI)	*p* value	NAD^+^/NADH ratio B (95% CI)	*p* value
HLY	−0.001 (−0.001–0.000)	0.003	0.084 (−0.183–0.351)	0.537	−0.006 (−0.013–0.001)	0.101
Age	0.001 (−0.002–0.004)	0.351	−1.534 (−2.866–−0.202)	0.024	0.012 (−0.023–0.047)	0.494
BMI	−0.002 (−0.007–0.004)	0.558	−0.864 (−3.258–1.529)	0.477	0.016 (−0.046–0.079)	0.607
Duration of disease	0.001 (−0.005–0.006)	0.819	1.760 (−0.864–4.384)	0.188	0.007 (−0.062–0.076)	0.838
HbA1c	0.000 (−0.026–0.027)	0.981	11.367 (−0.670–23.404)	0.064	0.069 (−0.246–0.385)	0.665
DNR	−0.724 (−0.841–−0.606)	< 0.001	−7.217 (−60.736–46.301)	0.791	−6.944 (−8.346–−5.542)	< 0.001
MNPDR	−0.741 (−0.890–−0.591)	< 0.001	114.850 (46.670–183.030)	0.001	−8.246 (−10.032–−6.460)	< 0.001
HRPDR	−0.979 (−1.154–−0.804)	< 0.001	161.878 (82.083–241.673)	< 0.001	−12.601 (−14.691–−10.512)	< 0.001
Sex (female)	−0.007 (−0.051–0.036)	0.746	−7.607 (−27.547–12.333)	0.453	−0.019 (−0.541–0.504)	0.944

*Note:* Data are presented as unstandardized regression coefficients (B) with 95% confidence intervals (CI) and p values derived from multivariate general linear modelling. HK, LDH, and NAD^+^/NADH were included as dependent variables. HLY was the primary predictor of interest. Models were adjusted for age, sex, BMI, HbA1c, duration of disease, and study group (entered as dummy variables with healthy controls (HC) as the reference category). Multivariate significance was assessed using Pillai’s Trace. A *p* value < 0.05 was considered statistically significant.

## 4. Discussion

The present study demonstrated significant changes in metabolic and redox parameters at different stages of DR, emphasizing the role of lipoxidative stress and metabolic dysregulation potentially associated with disease severity. There were no significant differences in demographic and clinical characteristics including age, sex, BMI, and BP across groups, indicating adequate matching and reducing potential confounding effects. However, diabetic groups had significantly higher values of glycemic parameters than healthy individuals, indicating persistent hyperglycemia as a major metabolic abnormality associated with diabetic status. Sustained hyperglycemia has been reported to cause oxidative stress through increased glucose auto‐oxidation, activation of the polyol pathway, and enhanced formation of advanced glycation and lipoxidation end products, which in turn contributes to cellular and microvascular damage [25, 26]. Nevertheless, the lack of major differences between diabetic subgroups suggests that hyperglycemia may be a common metabolic background in different stages of DR rather than a distinguishing marker of disease progression.

**FIGURE 4:**
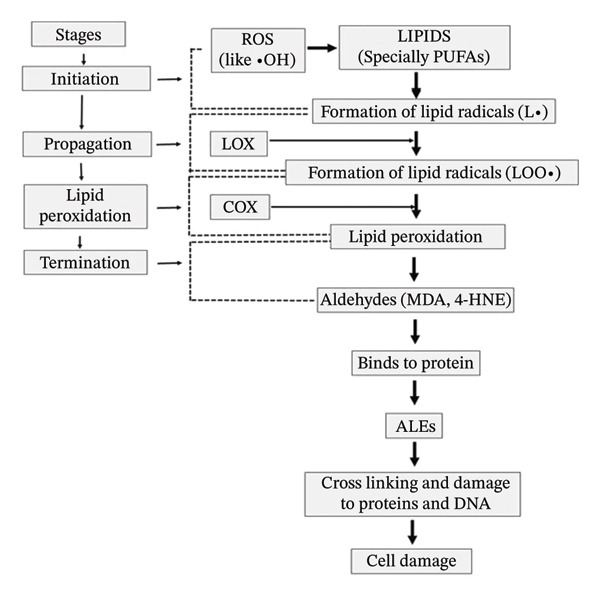
Schematic diagram of lipid peroxidation process. Abbreviations: ROS: reactive oxygen species, PUFAs: polyunsaturated fatty acids, L•: lipid radicals, LOO•: peroxyl radicals, LOX: lipoxygenase, COX: cyclooxygenase, MDA: malondialdehyde, 4‐HNE: 4‐hydroxy‐2‐nonenal, and ALE: advanced lipoxidation end product. Notes: Lipid peroxidation begins when ROS attack lipids, forming L•. These react with oxygen to form LOO•, propagating the chain reaction. LOX and COX enzymes facilitate this process. Lipid hydroperoxides break down into 4‐HNE and MDA, which react with proteins to form ALEs. These ALEs cause cross‐linking and damage to proteins and DNA, leading to cell damage.

A key finding was the progressive and significant increase in HLY levels across DR stages. HLY, a well‐recognized ALE component, showed a consistent rise from HC to HRPDR groups, with large effect sizes across comparisons. This supports the role of cumulative lipid peroxidation in DR progression rather than isolated oxidative events. Lipid peroxidation–derived ALEs promote platelet activation and aggregation while reducing nitric oxide (NO) bioavailability via endothelial dysfunction, thereby contributing to tissue hypoxia and microvascular damage. Previous studies have similarly reported elevated lipid peroxidation products in diabetic tissues, linking oxidative injury to structural and functional cellular impairment [2, 11]. The sequence of events leading to lipid peroxidation, ALE formation, and cellular damage is shown below as a flowchart (Figure 4).

In contrast, HK activity showed a progressive decline across disease stages, including between adjacent groups. As the rate‐limiting enzyme of glycolysis, HK is essential for glucose phosphorylation and initiation of the glycolytic pathway; therefore, reduced HK activity may indicate impaired glycolytic flux and metabolic inefficiency under oxidative stress conditions. Lipid peroxidation–derived aldehydes can modify lysine residues in proteins, thereby altering enzyme structure and function [2], [27]. Consistent with this mechanism, the ALE component HLY demonstrated a significant negative association with HK in the present study, suggesting a potential role of ALE in impairment of enzymatic activity. Moreover, the significantly lower HK activity observed in the MNPDR and HRPDR groups after adjustment for covariates further supports systemic alterations in glycolytic activity associated with increasing disease severity.

LDH activity increased progressively across all stages of DR. As a key enzyme of anaerobic glycolysis, LDH facilitates conversion of pyruvate to lactate. Its elevation may reflect a metabolic shift toward anaerobic glycolysis due to mitochondrial dysfunction and oxidative stress. Under impaired oxidative phosphorylation, cells rely more on anaerobic pathways for ATP production, resulting in increased lactate generation [28]. Similar metabolic adaptation has been observed in retinal cells under hypoxic and oxidative stress conditions [29]. This increase in LDH may suggest increased reliance on anaerobic metabolism as an alternative energy‐producing pathway under metabolic stress conditions.

Although LDH is a nonspecific marker of tissue injury, emerging evidence suggests that elevated systemic LDH reflects hypoxia‐induced metabolic reprogramming and may indicate progression from non‐proliferative to proliferative DR [30, 31]. However, because plasma LDH is a systemic biomarker, the elevated levels observed in the present study cannot be directly attributed to retinal tissue injury or retinal‐specific metabolic dysfunction. In line with this, our earlier research showed that higher plasma lactate, a byproduct of LDH‐driven anaerobic glycolysis, along with elevated vascular endothelial growth factor (VEGF) levels, indicates tissue hypoxia and hypoxia‐induced angiogenic processes [7]. Collectively, these markers may reflect hypoxia‐driven metabolic adaptation and retinal microvascular changes during disease progression. Therefore, elevated LDH in the present study should be interpreted as a systemic biochemical alteration associated with metabolic stress rather than a direct indicator of retinal tissue injury.

A further important observation was the progressive decline in the NAD^+^/NADH ratio, indicating worsening redox imbalance. NAD^+^ is essential for mitochondrial respiration, oxidative metabolism, and cellular repair. A reduced NAD^+^/NADH ratio is a well‐established marker of mitochondrial dysfunction and oxidative stress [32]. In diabetes, increased glucose flux elevates NADH production while reducing NAD^+^ availability, which may contribute to increased oxidative stress and ROS generation [33]. The observed decline, therefore, reflects persistent redox imbalance that may contribute to metabolic dysregulation in DR. These findings may indicate systemic alterations in redox‐related metabolic homeostasis associated with DR progression.

Consistent with these findings, post hoc analyses of HLY, HK, LDH, and NAD^+^/NADH ratio demonstrated significant stepwise differences with substantial to large effect sizes, suggesting that these biochemical alterations are both statistically and biologically relevant across DR stages.

Univariate linear regression analysis demonstrated that HLY was significantly associated with all three metabolic parameters, including HK, LDH, and NAD^+^/NADH ratio. The strongest associations were observed with HK and NAD^+^/NADH ratio, both showing high adjusted *R* ^2^ values, suggesting a close relationship between lipoxidative stress, impaired glycolytic initiation, and redox imbalance. These findings indicate that increasing HLY levels are associated with alterations in glycolytic entry, anaerobic metabolism, and oxidative stress status. Similar metabolic disturbances have previously been reported in diabetic complications [34].

Additionally, multivariate regression analysis (see Table 7) demonstrated a significant overall association between HLY and the combined metabolic outcomes, as indicated by Pillai’s Trace analysis. However, after adjustment for potential confounding variables, HLY remained independently associated only with HK, whereas associations with LDH and NAD^+^/NADH ratio became nonsignificant. This independent HLY–HK association represents the principal finding of the present study and suggests that lipoxidative stress may be more closely associated with glycolytic initiation than with other measured metabolic parameters. These findings suggest that impairment of glycolytic initiation may be associated with lipoxidative stress in DR, while alterations in anaerobic metabolism and redox balance may involve additional interacting metabolic mechanisms. Furthermore, age demonstrated an independent negative association with LDH levels, indicating that age‐related metabolic variation may also contribute to alterations in anaerobic glycolytic activity. The adjusted regression models for HK and NAD^+^/NADH ratio demonstrated high explanatory power, as reflected by the high adjusted *R* ^2^ values, indicating that the included metabolic and clinical variables collectively explain a substantial proportion of variability in these parameters.

From a pathophysiological standpoint, the observed pattern of increased HLY, decreased HK activity, increased LDH activity, and reduced NAD^+^/NADH ratio suggests a pattern of metabolic dysregulation that may be related to oxidative stress. Retinal tissue, due to its high metabolic demand, is particularly sensitive to disturbances in glycolysis and mitochondrial function [25, 35]. However, because the present study evaluated peripheral blood and erythrocyte biomarkers rather than retinal tissue or ocular fluid samples, these findings should not be interpreted as direct evidence of retinal‐specific metabolic dysfunction. These metabolic alterations may, therefore, represent systemic biochemical changes potentially associated with retinal dysfunction.

Clinically, HLY may serve as a marker of oxidative stress and disease severity, while reduced HK activity may reflect impaired glycolytic capacity and metabolic dysfunction. The persistence of the independent HLY–HK association after covariate adjustment further highlights HK as a potential metabolic target linked to lipoxidative injury in DR. Early detection of these changes may support further research into metabolic and oxidative pathways involved in DR. Antioxidant‐based interventions have previously shown potential in reducing oxidative injury and slowing diabetic complications [26, 36].

Although this study provides several valuable findings, the limitations are important to acknowledge. The cross‐sectional design restricts the ability to establish causal relationships between lipoxidative stress and metabolic alterations. In addition, the relatively limited sample size may affect the generalizability of the results to broader populations. Furthermore, the study was based exclusively on peripheral blood and erythrocyte‐derived biomarkers and did not include direct retinal tissue assessment, aqueous humor or vitreous humor analysis, or imaging‐based metabolic validation. Therefore, the observed biochemical alterations should be interpreted as systemic associations rather than direct evidence of retinal‐specific metabolic dysfunction. Therefore, future longitudinal and mechanistic studies are warranted to validate these findings and to further elucidate the underlying molecular interactions between lipoxidation and glycolytic dysregulation in DR.

## 5. Conclusion

In conclusion, the present study demonstrated significant associations between lipoxidative stress and metabolic alterations across different stages of DR. Among the evaluated metabolic parameters, HLY remained independently associated only with HK after multivariable adjustment, whereas its associations with LDH and NAD^+^/NADH ratio were no longer statistically significant. These findings suggest that lipoxidative stress may be more closely associated with alterations in glycolytic initiation than with other measured metabolic parameters. However, because the study was cross‐sectional and based on peripheral blood‐derived biomarkers, causal relationships cannot be established, and the findings should not be interpreted as direct evidence of retinal‐specific metabolic dysfunction. Nevertheless, these observations provide supportive evidence for future studies exploring systemic metabolic and oxidative alterations associated with DR.

## Author Contributions

Anwesa Mondal and Subhasish Pramanik: perform laboratory experiments, collection of data, software, data analysis, and writing–original draft. Chiranjit Bose: writing–original draft and formal analysis. Asif Mondal: writing–original draft. Lakshmi Kanta Mondal: conceptualization, methodology, and writing–reviewing and editing.

## Funding

This study did not receive any specific grants from funding agencies in the public, commercial, or not‐for‐profit sectors.

## Disclosure

Parts of the present study were previously published as a preprint in 2024, available at https://www.preprints.org/manuscript/202411.1208. All authors contributed to the article and approved the submitted version.

## Ethics Statement

This study was approved by the Institutional Ethics Committee (Medical College, Kolkata, Ref. No: MC/KOL/IEC/NON‐SPON/181/12–2018). Informed consent was obtained from all patients according to the Declaration of Helsinki.

## Conflicts of Interest

The authors declare no conflicts of interest.

## Data Availability

The datasets generated and/or analyzed during the current study are available from the corresponding author upon reasonable request.

## References

[1] The Diabetes Control and Complications Trial Research Group , Diabetes Control and Complications Trial (DCCT): Results of Feasibility Study, Diabetes Care. (1987) 10, no. 1, 1–19, 10.2337/diacare.10.1.1. 2882967

[2] Mondal L. K. , Pramanik S. , Choudhuri S. et al., Do Different Lipid Components Accelerate the Pathogenesis and Severity of Diabetic Retinopathy?, International Journal of Retina and Vitreous. (2022) 8, no. 1, 10.1186/s40942-022-00390-4. PMC9188217 35690853

[3] Choudhuri S. , Mondal L. K. , Paine S. K. et al., Role of Hyperglycemia-Mediated Erythrocyte Redox State Alteration in the Development of Diabetic Retinopathy, Retina. (2013) 33, no. 1, 207–216, 10.1097/iae.0b013e318256202e. 22653543

[4] Chaudhry R. and Varacallo M. A. , Biochemistry, Glycolysis, Statpearls. Treasure Island (FL), 2023, StatPearls Publishing. 29493928

[5] Berg J. M. , Tymoczko J. L. , Gatto G. J.Jr, and Stryer L. , Glycolysis and the Catabolism of Hexoses, Biochemistry, 2015, 8th edition, W. H. Freeman, New York.

[6] Rabbani N. , Xue M. , and Thornalley P. J. , Hexokinase-2-Linked Glycolytic Overload and Unscheduled Glycolysis—Driver of Insulin Resistance and Development of Vascular Complications of Diabetes, International Journal of Molecular Sciences. (2022) 23, no. 4, 10.3390/ijms23042165. PMC8877341 35216280

[7] Mondal L. K. , Bhaduri G. , and Bhattacharya B. , Biochemical Scenario Behind Initiation of Diabetic Retinopathy in Type 2 Diabetes Mellitus, Indian Journal of Ophthalmology. (2018) 66, no. 4, 535–540, 10.4103/ijo.IJO_884_17. 29582815 PMC5892057

[8] Billups B. and Attwell D. , Modulation of Non-Vesicular Glutamate Release by pH, Nature. (1996) 379, no. 6561, 171–174, 10.1038/379171a0. 8538768

[9] Gusev E. I. and Skvortsova V. I. , The Glutamate–Calcium Cascade, Brain Ischemia, 2003, Springer, Boston.

[10] Macdonald R. L. and Weir B. K. , Cerebral Vasospasm and Free Radicals, Free Radical Biology and Medicine. (1994) 16, no. 5, 633–643, 10.1016/0891-5849(94)90064-7. 8026807

[11] Pamplona R. , Advanced Lipoxidation End-Products, Chemico-Biological Interactions. (2011) 192, no. 1–2, 14–20, 10.1016/j.cbi.2011.01.007. 21238437

[12] Choudhuri S. , Dutta D. , Chowdhury I. H. et al., Association of Hyperglycemia-Mediated Increased Advanced Glycation and Erythrocyte Antioxidant Enzyme Activity in Different Stages of Diabetic Retinopathy, Diabetes Research and Clinical Practice. (2013) 100, no. 3, 376–384, 10.1016/j.diabres.2013.03.031. 23602454

[13] Choudhuri S. , Roy P. K. , Mitra B. et al., Hyperlipidemia-Mediated Increased Advanced Lipoxidation End Products Formation, an Important Factor Associated With Decreased Erythrocyte Glucose-6-Phosphate Dehydrogenase Activity in Mild Nonproliferative Diabetic Retinopathy, Canadian Journal of Diabetes. (2017) 41, no. 1, 82–89, 10.1016/j.jcjd.2016.07.007. 27916496

[14] Mayer J. and Donnelly T. M. , Glucose, Clinical Veterinary Advisor, 2013, W.B. Saunders, St Louis.

[15] Khan H. A. , Alhomida A. S. , Sobki S. H. et al., Serum Markers of Tissue Damage and Oxidative Stress in Patients With Acute Myocardial Infarction, Biomedical Research. (2013) 24, no. 1, 15–20.

[16] Drent M. , Cobben N. A. , Henderson R. F. , Wouters E. F. , and van Dieijen-Visser M. , Usefulness of Lactate Dehydrogenase and Its Isoenzymes as Indicators of Lung Damage or Inflammation, European Respiratory Journal. (1996) 9, no. 8, 1736–1742, 10.1183/09031936.96.09081736. 8866602

[17] Herman W. H. , Pop-Busui R. , Braffett B. H. et al., Use of the Michigan Neuropathy Screening Instrument as a Measure of Distal Symmetrical Peripheral Neuropathy in Type 1 Diabetes: Results From the Diabetes Control and Complications Trial/Epidemiology of Diabetes Interventions and Complications, Diabetic Medicine. (2012) 29, no. 7, 937–944, 10.1111/j.1464-5491.2012.03644.x. 22417277 PMC3641573

[18] Coskun M. and Sevencan N. O. , The Evaluation of Ophthalmic Findings in Women Patients With Iron and Vitamin B12 Deficiency Anemia, Translational Vision Science & Technology. (2018) 7, no. 4, 10.1167/tvst.7.4.16. PMC6108531 30159209

[19] Pramanik S. , Chowdhury S. , Ganguly U. , Banerjee A. , Bhattacharya B. , and Mondal L. K. , Visual Contrast Sensitivity Could be an Early Marker of Diabetic Retinopathy, Heliyon. (2020) 6, no. 10, 10.1016/j.heliyon.2020.e05336. PMC7591734 33145449

[20] Hoffman R. , Brook G. J. , Aviram M. et al., Hypolipidemic Drugs Reduce Lipoprotein Susceptibility to Undergo Lipid Peroxidation: In Vitro and Ex Vivo Studies, Atherosclerosis. (1992) 93, no. 1–2, 105–113, 10.1016/0021-9150(92)90204-t. 1596293

[21] Broncel M. , Koter-Michalak M. , and Chojnowska-Jezierska J. , The Effect of Statins on Lipid Peroxidation and Activities of Antioxidant Enzymes in Patients With Dyslipidemia, Przeglad Lekarski. (2006) 63, no. 9, 738–742. 17479860

[22] Early Treatment Diabetic Retinopathy Study Design and Baseline Patient Characteristics. ETDRS Report Number 7, Ophthalmology. (1991) 98, no. 5 Suppl, 741–756, 10.1016/s0161-6420(13)38009-9. 2062510

[23] Ramalingam S. , Karuppiah M. , Thiruppathi M. , Palanivelu S. , and Panchanatham S. , Antioxidant Potential of Biflavonoid Attenuates Hyperglycemia by Modulating the Carbohydrate Metabolic Enzymes in High Fat Diet/Streptozotocin-Induced Diabetic Rats, Redox Report. (2020) 25, no. 1, 1–10, 10.1080/13510002.2020.1722914. 32013797 PMC7034448

[24] Javaraiah R. K. , David C. M. , Namitha J. , Tiwari R. , and Benakanal P. , Evaluation of Salivary Lactate Dehydrogenase as a Prognostic Biomarker in Tobacco Users With and Without Potentially Malignant Disorders of the Oral Cavity, South Asian Journal Cancer. (2020) 9, no. 2, 93–98, 10.1055/s-0040-1721174. PMC7745746 33354552

[25] Hammes H. P. , Diabetic Retinopathy: Hyperglycaemia, Oxidative Stress and Beyond, Diabetologia. (2018) 61, no. 1, 29–38, 10.1007/s00125-017-4435-8. 28942458

[26] Forbes J. M. and Cooper M. E. , Mechanisms of Diabetic Complications, Physiological Reviews. (2013) 93, no. 1, 137–188, 10.1152/physrev.00045.2011. 23303908

[27] A , Muñoz M. F. , and Argüelles S. , Lipid Peroxidation Mechanisms, Oxidative Medicine and Cellular Longevity. (2014) 2014. 10.1155/2014/360438 PMC4066722 24999379

[28] Liberti M. V. and Locasale J. W. , The Warburg Effect, Trends in Biochemical Sciences. (2016) 41, no. 3, 211–218. 26778478 10.1016/j.tibs.2015.12.001 PMC4783224

[29] Punzo C. , Kornacker K. , and Cepko C. L. , Metabolic Pathways in Retinal Degeneration, Nature Neuroscience. (2009) 12, no. 1, 44–52. 19060896 10.1038/nn.2234 PMC3339764

[30] Wu Y. , Lu C. , Pan N. et al., Serum LDH Activities as Biomarkers, Scientific Reports. (2021) 11. 10.1038/s41598-021-92430-6 PMC8217520 34155288

[31] Shosha E. , Chauhan M. Z. , Muayad J. , Sallam A. B. , and Fouda A. Y. , Systemic LDH and DR Progression, Journal of Clinical Medicine. (2025) 14, no. 24. 10.3390/jcm14248696 PMC12733795 41464598

[32] Ying W. , NAD^+^/NADH in Cellular Functions, Frontiers in Bioscience. (2006) 11, 3129–3148. 16720381 10.2741/2038

[33] Du X. , Matsumura T. , Edelstein D. et al., Hyperglycemic Damage Pathways, Journal of Clinical Investigation. (2003) 112, no. 7, 1049–1057. 14523042 10.1172/JCI18127 PMC198524

[34] Kowluru R. A. and Chan P. S. , Oxidative Stress and DR, Experimental Diabetes Research. (2007) 2007. 10.1155/2007/43603 PMC1880867 17641741

[35] Kang Q. and Yang C. , Oxidative Stress and Diabetic Retinopathy: Molecular Mechanisms, Pathogenetic Role and Therapeutic Implications, Redox Biology. (2020) 37, 10.1016/j.redox.2020.101799. PMC7767789 33248932

[36] Kowluru R. A. and Mishra M. , Oxidative Stress, Mitochondrial Damage and Diabetic Retinopathy, Biochimica Et Biophysica Acta. (2015) 1852, no. 11, 2474–2483, 10.1016/j.bbadis.2015.08.001. 26248057

